# Antiproliferative Efficacy of *N*-(3-chloro-4-fluorophenyl)-6,7-dimethoxyquinazolin-4-amine, DW-8, in Colon Cancer Cells Is Mediated by Intrinsic Apoptosis

**DOI:** 10.3390/molecules26154417

**Published:** 2021-07-22

**Authors:** Rabin Neupane, Saloni Malla, Mariam Sami Abou-Dahech, Swapnaa Balaji, Shikha Kumari, Digambar Kumar Waiker, N. S. Hari Narayana Moorthy, Piyush Trivedi, Charles R. Ashby, Chandrabose Karthikeyan, Amit K. Tiwari

**Affiliations:** 1 Department of Pharmacology and Experimental Therapeutics, College of Pharmacy and Pharmaceutical Sciences, The University of Toledo, Health Science Campus, 3000 Arlington Ave, Toledo, OH 43614, USA; rabin.neupane@rockets.utoledo.edu (R.N.); saloni.malla@rockets.utoledo.edu (S.M.); mariam.aboudahech@rockets.utoledo.edu (M.S.A.-D.); swapnaa.balaji@rockets.utoledo.edu (S.B.); shikha.kumari@utoledo.edu (S.K.); 2 School of Pharmaceutical Sciences, Rajiv Gandhi Proudyogiki Vishwavidyalaya, Bhopal 462033, India; digamber35@gmail.com; 3 Department of Pharmacy, Indira Gandhi National Tribal University, Lalpur, Amarkantak 484887, India; nshnarayanamoorthy@gmail.com; 4 Center of Innovation and Translational Research, Poona College of Pharmacy, Bharati Vidyapeeth Deemed University, Pune 411030, India; piyushtrivedi304@gmail.com; 5 Department of Pharmaceutical Sciences, College of Pharmacy, St. John’s University, Queens, NY 11439, USA; cnsratdoc@optonline.net; 6 Department Centre of Medical and Bio-allied Health Sciences Research (CMBHSR), Ajman University, Ajman P.O. Box 346, United Arab Emirates; 7 Department of Cancer Biology, College of Medicine and Life Sciences, The University of Toledo, Health Science Campus, 3000 Arlington Ave, Toledo, OH 43614, USA

**Keywords:** colorectal cancer, cytotoxicity, 4-anilino-quinazoline, intrinsic apoptosis, anticancer compound

## Abstract

A novel series of 4-anilinoquinazoline analogues, **DW (1–10)**, were evaluated for anticancer efficacy in human breast cancer (BT-20) and human colorectal cancer (CRC) cell lines (HCT116, HT29, and SW620). The compound, **DW-8**, had the highest anticancer efficacy and selectivity in the colorectal cancer cell lines, HCT116, HT29, and SW620, with IC_50_ values of 8.50 ± 2.53 µM, 5.80 ± 0.92 µM, and 6.15 ± 0.37 µM, respectively, compared to the non-cancerous colon cell line, CRL1459, with an IC_50_ of 14.05 ± 0.37 µM. The selectivity index of **DW-8** was >2-fold in colon cancer cells incubated with vehicle. We further determined the mechanisms of cell death induced by **DW-8** in SW620 CRC cancer cells. **DW-8** (10 and 30 µM) induced apoptosis by (1) producing cell cycle arrest at the G2 phase; (2) activating the intrinsic apoptotic pathway, as indicated by the activation of caspase-9 and the executioner caspases-3 and 7; (3) nuclear fragmentation and (4) increasing the levels of reactive oxygen species (ROS). Overall, our results suggest that **DW-8** may represent a suitable lead for developing novel compounds to treat CRC.

## 1. Introduction

Colorectal cancer (CRC) is the third most common malignancy in the world and approximately 1.9 million new cases and 935,173 deaths were recorded in 2020 [1]. Despite the rapid advances in the detection and diagnosis of colorectal cancer, the prognosis for advanced or metastatic CRC is still very poor [2]. Palliative chemotherapeutic regimens, consisting of fluoropyrimidines, in combination with leucovorin and either oxaliplatin (FOLFOX) or irinotecan (FOLFIRI), are still the first-line treatment for patients with advanced and metastatic CRC [3]. However, these chemotherapeutic regimens can become ineffective due to the development of multidrug resistance (MDR) [4]. Therefore, there is a critical need to develop a novel drug to treat advanced and metastatic colorectal cancer.

The quinazoline ring is considered an important pharmacophore in medicinal chemistry for the discover and develop novel anticancer compounds [5]. Moreover, the quinazoline derivatives have anticancer efficacy in multiple cancer types by different mechanisms of action [6,7,8]. A number of 4-anilinoquinazolines derivatives inhibit certain protein kinases by competitively and reversibly inhibiting ATP binding at the ATP catalytic site [9,10]. Currently, several quinazoline-based drugs, such as gefitinib, erlotinib, vandetanib, lapatinib, and afatinib, are used clinically to treat cancer. [5]. Interestingly, several small molecules are present in clinical development pipeline also contain quinazoline moiety [11], suggesting that it may serve as an important scaffold to discover potent anticancer agents. Inspired by these findings, our group has resynthesized and evaluated various anilinoquinazolines analogues for their *in vitro* anticancer potential in human breast and colon cancer cells.

Apoptosis is a programmed cell death mechanism activated by the majority of chemotherapeutic compounds [12]. Based on the molecular mechanism, apoptosis can be classified as either intrinsic or extrinsic [12]. In the intrinsic apoptosis pathway, the proteins, Bcl2-antagonist/killer 1 (BAK) and Bcl2 associated X (BAX) interact with the mitochondrial membrane, forming pores, thereby facilitating the release of mitochondrial membrane proteins, such as cytochrome c, into the cytoplasm [13,14]. The anti-apoptotic protein, B-cell lymphoma 2 (Bcl2) is downregulated in cancer cells, thereby decreasing the likelihood of apoptosis [13]. Furthermore, apoptosis can be induced by the activation of caspase 9, an initiator caspase, followed by activation of the executioner caspases-3 and 7. The activation of caspase-3 and 7 induces cleavage and biotransformation of a number of cellular substrates, which ultimately produces blebbing, disassembly of cellular structures, and DNA fragmentation [15]. Cleaved caspase-3 can activate poly (ADP-ribose) polymerase (PARP), which will decrease the extent of poly(ADP-ribosyl)ation of chromatin-bound Ca^2+^/Mg^2+^-dependent endonuclease, leading to the disinhibition of DNasel-Like III protein (DNASIL3), producing biodegradation of genomic DNA [16]. Here, we have probed for different apoptosis markers in CRC cells incubated with **DW-8** to establish apoptosis as a mechanism of cell death induced by **DW-8**.

## 2. Results

### 2.1. Chemistry

**Scheme 1:**
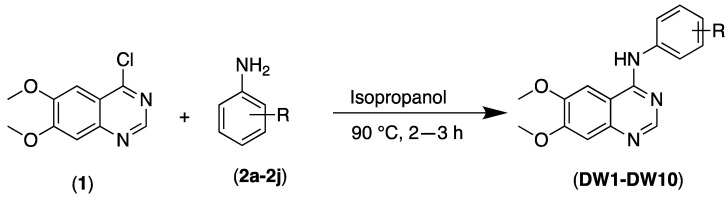
Synthesis of 4-anilinoquinazolines (**DW1-DW10**).

The 6,7-dimethoxy-*N*-phenylquinazolin-4-amines (**DW 1–10**) synthesis in high yields by reacting 4-chloro-6,7-dimethoxy quinazoline (1), with the corresponding substituted anilines substrates (2), under the conditions as shown in Scheme 1, has been previously reported [17].

### 2.2. 3-(4,5-Dimethylthiazol-2-yl)-2,5-Diphenyltetrazolium Bromide-Based Cytotoxicity Assay and Morphological Changes Induced by ***DW-8*** in SW620 Cells

The synthesized compounds (**DW 1**–**10**) were initially evaluated for their anticancer activity in the human breast cancer cell line, BT-20 and the human colorectal cancer cell line, HCT116, using the 3-(4,5-dimethylthiazol-2yl)-2,5-diphenyltetrazolium bromide (MTT) colorimetric assay, by incubating the cells with **DW** (**1**–**10**), at 1, 10, and 100 µM. Table 1 shows the IC_50_ values of compounds (**DW 1**–**10**) for each cell line obtained from the MTT assay.

**Figure 1:**
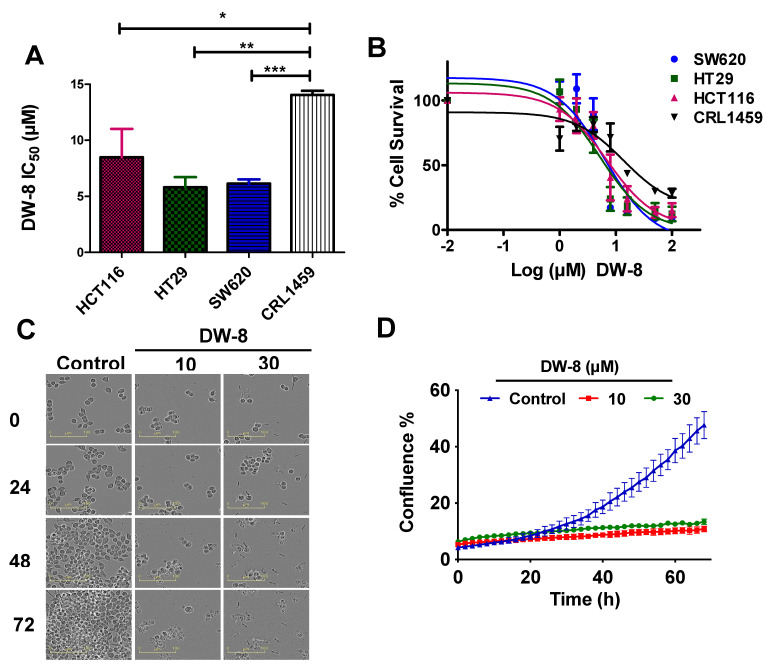
The anti-proliferative efficacy of compound **DW-8** in different colon cancer cell lines. (**A**) The IC_50_ values for **DW**-**8** in HCT116, HT29, SW620, and CRL1459 cells after 72 h of incubation. (**B**) The cell viability curves for HCT116, HT29, SW620 and CRL1459 cells that were incubated with different concentrations (1–100 µM) of **DW-8** over 72 h. (**C**) Changes in SW620 cells morphology after incubation with vehicle (negative control), 10 and 30 µM of **DW-8**, at different time points (0, 24, 48, and 72 h). (**D**) A representative confluence graph of SW620 cells incubated with vehicle (negative control), 10 and 30 µM of **DW-8**. The data represent the mean ± SD of three independent experiments performed in triplicate. * *p* < 0.05, ** *p* < 0.01, *** *p* < 0.001.

Our results indicated that the compound, **DW-8**, was the most potent analogue among all tested analogues in inhibiting the proliferation of HCT116 and BT-20 cancer cells. Additionally, we also determined the efficacy of **DW-8** in the human colorectal adenocarcinoma cell lines, HT-29 and SW620 and in human non-cancerous colon cell line, CRL1459. The results indicated that the IC_50_ values for **DW-8** in the HT-29, SW620, and CRL1459 cells were 5.80 ± 0.92 µM, 6.15 ± 0.37 µM, and 14.05 ± 0.37 µM, respectively (Figure 1A). Interestingly, the IC_50_ of HCT116, HT-29, and SW620 cell lines were significantly lower that that for the CRL1459 cells, with *p*-values of 0.0326, 0.0014, and 0.0002, respectively, when compared to CRL1459. All cells lines (i.e., HCT116, HT-29, SW620, and CRL1459) incubated with **DW-8** for 72 h showed a concentration-dependent decrease in the viability (Figure 1B). Further studies were conducted in the SW620 cell line, which had a low IC_50_ value and the lowest standard deviation, compared to HT-29 and HCT116 cells. 

Morphologically, apoptosis is characterized by cell shrinkage, nuclear fragmentation, chromosome condensation, extensive blebbing of the plasma membrane and the formation of apoptotic bodies [18]. The incubation of SW620 cells with **DW-8** produced apoptosis (Figure 1C). **DW-8**, at 10 and 30 µM, significantly decreased the number of viable and adherent SW620 cells, compared to cells incubated with vehicle (negative control) (Figure 1C). The SW620 cells shrank in size and became round in shape. The majority of cells were floating and other cells were loosely adhered on the surface. In addition, the presence of apoptotic bodies occurred at 10 and 30 µM of **DW-8** (Figure 1C). In contrast, the negative control cells were viable, non-apoptotic and replicated over time, reaching maximum confluence after 72 h of incubation (Figure 1D).

### 2.3. The Effect ***DW-8*** on the Cell Cycle and Mitochondrial Membrane Potential of SW620 Cells

**Figure 2:**
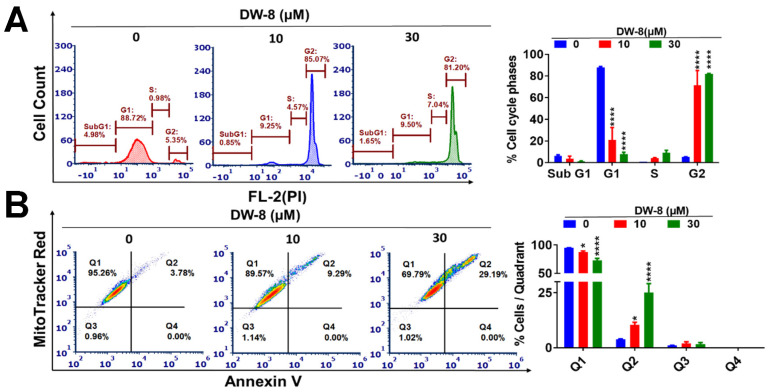
The effect of **DW-8** on the apoptosis and cell cycle phases of SW620 cells. (**A**) A representative figure showing the effect of **DW-8** on the phases of the cell cycle in SW620 cells. A histogram quantitatively summarizing the change in the percentage of cells in each phase of the cell cycle after incubation with **DW-8**. (**B**) A representative figure showing the induction of apoptosis by vehicle, 10 or 30 µM of **DW-8**, based on data obtained using the MitoTracker Red and Alexa Fluor 488 Annexin kits. A histogram summarizing the same results is also shown; * *p* < 0.05, **** *p* < 0.0001. The data represent means ± SD of three independent experiments performed in triplicate. FL-2 indicates the gate used in a flow cytometer. PI is the abbreviation for Propidium iodide.

The majority of clinically used chemotherapeutic drugs elicit their therapeutic efficacy by inducing cancer cell apoptosis [19,20]. Anticancer drugs can produce efficacy by targeting the cell cycle regulating signaling pathways [21]. Therefore, we determined the effect of **DW-8** in the cell cycle of SW620 cell line. The incubation of SW620 cells with vehicle yielded the following distributions in the different phases of the cell cycle: 4.98% in SubG1, 88.72% in G1, 0.98% in S, and 5.35% in G2 (Figure 2A). After the incubation of SW620 cells with 10 and 30 µM of **DW-8**, the percentage of cells in the G1 phase was significantly decreased to 9.25 and 9.50%, respectively (*p* < 0.001). The incubation of SW620 cells with 10 and 30 µM significantly increased the percentage of cells in the G2 phase (Figure 2A). At 10 and 30 µM, **DW**-**8** produced an 85.07% (*p* < 0.001) and 81.20% (*p* < 0.001) increase, respectively, in the number of SW620 cells in the G2 phase of the cell cycle, i.e., the cell cycle was arrested in the G2 phase (Figure 2A).

One of the major events that occurs in apoptotic cells is the translocation of phosphatidyl serine (PS) from the inner side of the plasma membrane to the outer membrane [22]. Phosphatidyl serine is a Ca^2+^-dependent phospholipid binding protein that has high binding affinity and specificity for the protein, Annexin V, which is an inhibitor of prothrombin activation [23]. Therefore, apoptotic cells can be detected using fluorophore-labelled Annexin V [24]. As indicated in Figure 2B, the incubation of cells with 10 and 30 µM of **DW-8** produced a concentration-dependent increase in Annexin V staining. It is indicated by the increased number of cells in the second quadrant (Q2) in **DW-8** treated cells, compared to the negative control, indicating an increase in apoptosis (Figure 2B).

We also determined the effect of **DW-8** on the mitochondrial membrane potential in SW620 cells using MitoTracker^®^ Red dye. The dye stains the mitochondria in live cells and its accumulation depends on an intact or normal membrane potential [25]. The majority of SW620 cells incubated with the vehicle (negative control) were viable and located in quadrant I (95.26%), with only a few apoptotic cells in quadrant II (3.78%) (Figure 2B). In contrast, the incubation of SW620 cells with 10 and 30 µM of **DW-8** significantly increased the number of apoptotic cells in quadrant II, 9.29% (*p* < 0.5) and 29.19% (*p* < 0.01), respectively, compared to the vehicle (Figure 2B). This shift towards quadrant II suggests that the loss of mitochondrial membrane potential can induce apoptosis (Figure 2B).

### 2.4. ***DW-8*** Increases the Induction of Apoptosis by Activating the Intrinsic Apoptotic Pathway

**Figure 3:**
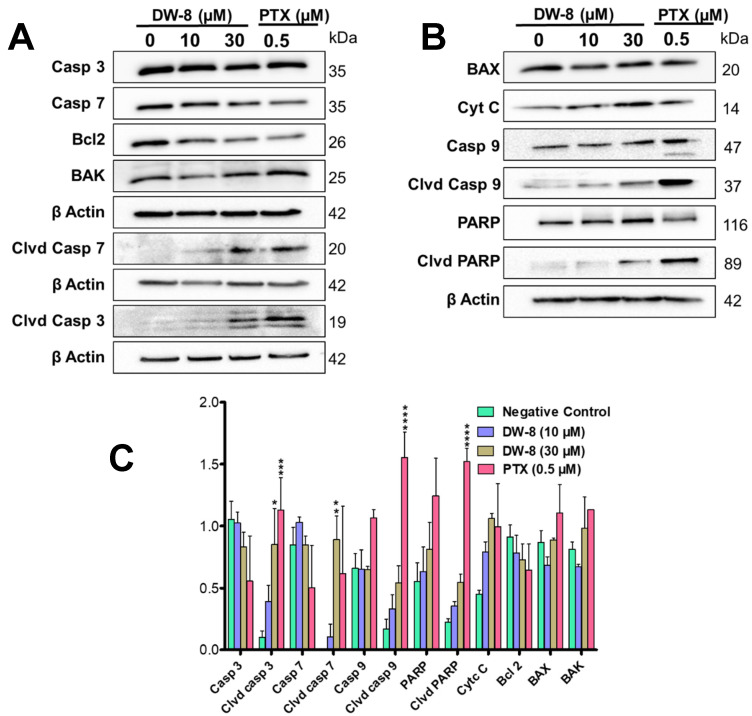
The induction of apoptosis in SW620 cells by **DW-8**. Figure (**A**,**B**) showing the western blots for the proteins involved in the intrinsic apoptotic pathway caspase-3, caspase-7, caspase-9, cleaved caspase-3, cleaved caspase-7, cleaved caspase caspase-9, Bcl2, BAK, BAX, PARP, cleaved PARP, and cytochrome c from lysates of SW620 cells incubated overnight with the vehicle (Control), 10 and 30 µM of **DW-8** and 0.5 µM of paclitaxel (a positive control). Each protein in the blot was normalized to β-actin level. (**C**) A histogram summarizing the proteins quantified from the western blots using Image J. The data represent means ± SD of two independent experiments with * *p* < 0.05, ** *p* < 0.01, *** *p* < 0.001, **** *p* < 0.0001. Clvd, Casp, PTX are abbreviations used for cleaved caspase, and paclitaxel, respectively.

The key regulators of apoptosis, such as caspases (3, 7, and 9), BAX, BAK, Bcl-2, PARP and cytochrome c, were determined in the lysates of SW620 cells incubated with **DW-8** (10 and 30 μM) and paclitaxel (0.5 µM, a positive control). The level of cytochrome c was significantly increased in the lysates of SW620 cells incubated with **DW-8** and the positive control, compared to the negative control (0 µM **DW-8**) (Figure 3B,C). The expression of Bcl2 decreased with an increasing concentration of **DW-8** (Figure 3A,C). Its expression was low in the positive control, compared to the negative control (Figure 3A,C). Although there was no significant change in the levels of BAX and BAK, the level of cleaved caspase-9 was significantly increased in cell lysates of SW620 cells incubated with **DW-8** and paclitaxel, compared to the negative control, suggesting that the intrinsic apoptotic pathway was activated (Figure 3A,B). Furthermore, cleaved caspase-3 (*p* < 0.05) and cleaved caspase-7 (*p* < 0.01) were significantly upregulated in the lysates of Sw620 incubated with **DW-8** and paclitaxel, compared to the negative control, suggesting the induction of caspases-dependent apoptosis by **DW-8** (Figure 3A,C). **DW-8** produced a concentration-dependent increase in the levels of cleaved PARP compared to the negative control. The expression of cleaved PARP was significantly greater in lysates of SW620 incubated with 0.5 µM of paclitaxel, compared to the negative control (Figure 3B,C).

### 2.5. Nuclear Morphology and Reactive Oxygen Species (ROS) Assay

**Figure 4:**
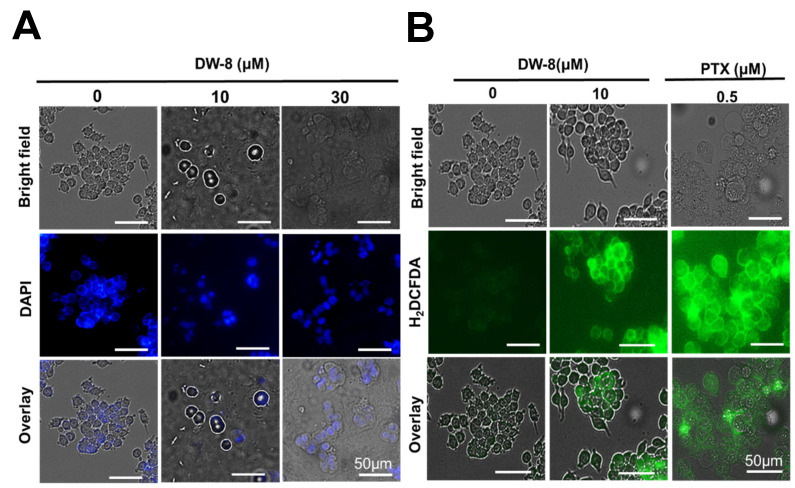
(**A**) The effect of the incubation of SW620 cells with the vehicle (0 µM), 10 and 30 µM of **DW-8**, followed by DAPI, on cell nuclear morphology. The nuclei of the cells incubated with the vehicle were circular and intact, whereas the nuclei of cells incubated with 10 and 30 µM of **DW-8** were shrunken and fragmented. (**B**) The effect of vehicle (0 µM **DW-8**), 10 µM of **DW-8** and 0.5 µM of paclitaxel (PTX) on the fluorescence level of the compound DCFDA, due to the biotransformation of H_2_DCFDA by cellular esterases. The level of green fluorescence and thus, the levels of ROS, produced by **DW-8** and paclitaxel, was significantly greater (*p*-value < 0.0001) than the negative control. The data represent the means ± SD of two independent experiments.

Nuclear morphological changes in SW620 cells were visualized after incubating cells with the nuclear staining dye, 4′, 6-diamidino-2-phenylindole dichloride (DAPI), followed by fluorescence microscopy. DAPI can enter living cells and stain the nucleus by binding strongly to adenine-thymine-rich regions of DNA, producing a blue fluorescence [26]. The nuclei of SW620 cells incubated with the vehicle (0 µM **DW-8**) were intact and circular in shape, whereas the nuclei of the SW620 cells incubated with 10 and 30 µM of **DW-8** were condensed and fragmented (Figure 4A). These results suggest that the SW620 cells incubated with **DW-8** underwent apoptosis and showed nuclear condensation, followed by DNA fragmentation, which are the hallmark morphological changes that occur during apoptosis [18,27].

Under reactive oxygen species (ROS) stress, the p53 enzyme system can upregulate apoptosis by promoting the transcription of pro-apoptotic proteins (e.g., BAX, BAK) and downregulating the transcription of pro-survival proteins (e.g., Bcl-2, Bcl-X_L_, and Mcl-1) [28]. Furthermore, p53 can increase mitochondrial membrane permeabilization, causing the release pro-apoptotic molecules, cytochrome c, and ROS, further increasing the likelihood of cell death [29]. Therefore, we determined the effect of **DW-8** on the levels of ROS in SW620 cells. This was done using the compound, 2, 7-dichlorodihydrofluorescein diacetate (H_2_DCFDA), where the diacetate groups are hydrolyzed by esterase enzymes after entering the cells, producing H_2_DCF [30]. Subsequently, the ROS present in cells can oxidize the H_2_DCF into 2′7′ dichlorofluorescein (DCF), which is a highly fluorescent dye [30]. The incubation of SW620 cells with **DW-8** (10 µM) and paclitaxel (0.5 µM) produced a significantly greater level of fluorescence compared to the negative control (vehicle; *p*-value < 0.0001) (Figure 4B). As previously reported [31,32], the incubation of cancer cells with paclitaxel produces a significantly greater level of ROS compared to the negative control (vehicle). Overall, these results indicate that **DW-8** significantly increases the level of ROS, which could facilitate cellular apoptosis.

### 2.6. The Effect of ***DW-8*** on Cell Viability Using the Incucyte™ Cytotox Green Assay

**Figure 5:**
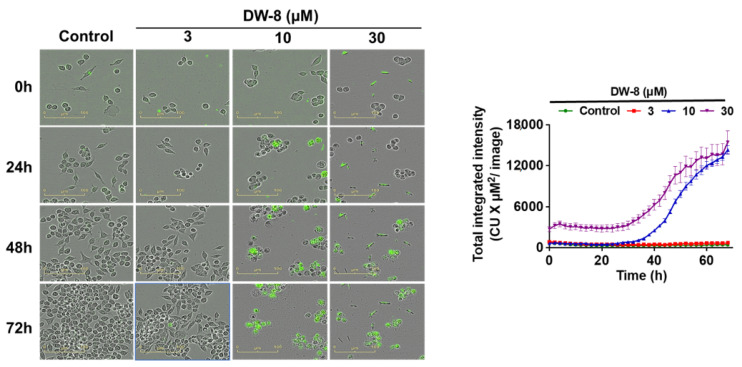
Incucyte images of SW620 cells incubated with the vehicle (negative control), 3, 10, and 30 µM of **DW-8** for 72 h, followed by staining with Incucyte Cytotox Green. The green fluorescence was significantly increased by 10 and 30 µM of **DW-8**, compared to cells incubated with the vehicle or 3 µM of **DW-8**. The data represent means ± SD of three independent experiments performed in triplicate.

In this assay, we determined the effect of the vehicle and **DW-8** on the level of fluorescence after incubation with the Incucyte™ Cytotox green reagent, a cyanine nucleic acid dye that has a high affinity to DNA and is used as a marker for dead cells as it only penetrates cells that have a disrupted or compromised cell membrane [33]. The fluorescence level in SW620 cells incubated with 3 µM of **DW-8** for 72 h did not significantly change, compared to the vehicle (negative control). In contrast, the level of fluorescence was significantly increased in SW620 cells after incubation with 10 and 30 µM of **DW-8** for 72 h, compared to the negative control (Figure 5).

## 3. Discussion and Conclusions

In this study, a series of 4-anilino quinazoline derivatives were evaluated for their in vitro anti-proliferation efficacy in a breast cancer cell line, BT-20 and the colorectal cancer cell lines, HCT116, HT29, and SW620. One compound, **DW-8**, had the highest anti-cancer efficacy in the CRC cell lines, compared to other analogues in the DW series. Upon investigating the mechanism by which **DW-8** produce cytotoxicity, our results indicate that **DW-8** induced cell death by activating the intrinsic apoptotic pathway. Apoptosis is a cell death pathway that is induced by the majority of clinically available chemotherapeutic drugs [20,34]. Moreover, a number of new molecules investigated for anti-cancer potential have been reported to produce cell death by inducing apoptosis [35]. Cancer progression can result from the unregulated expression of cyclin or cyclin-dependent kinases (CDKs), which impairs the cell cycle [36]. Therefore, cyclin and CDKs inhibitor can be useful in the management of cancer [36]. Our results indicated cell cycle arrest in the G2 phase in SW620 cells incubated with **DW-8**. It would be interesting to further investigate the role of **DW-8** in the inhibition of cyclin and CDKs. The morphological studies indicated that **DW-8** produced cell shrinkage and nuclear condensation, followed by nuclear fragmentation. Furthermore, DAPI staining indicated the presence of condensed nuclei and fragments of nuclei in SW620 cells incubated with **DW-8**. These morphological changes, such as nuclear condensation, cell shrinkage, blebbling and apoptotic bodies formation, are hallmark morphological changes in apoptosis [18]. The ROS level in **DW-8**-incubated cells was significantly increased compared to the negative control. ROS are involved in the release of cytochrome c from mitochondria into the cytoplasm where it can activate certain caspases and induce apoptosis [37]. Here, our results indicated an increase in ROS levels, followed by an increase in cytochrome c expression in SW620 cells incubated with **DW-8,** compared to the negative control. Overall, our results suggest that **DW-8** could be a suitable lead for developing novel compounds to treat CRC.

In addition to CRC, **DW-8** efficacy should be evaluated in in other cancer cell lines. Further studies should be conducted to ascertain if **DW-8** inhibits cancer cell proliferation by other mechanisms, such as inhibiting certain protein kinases.

## 4. Material and Methods

### 4.1. Chemistry

The procedure for the synthesis and the analytical data for the compounds (**DW-1** to **DW-10**) is reported elsewhere [17].

### 4.2. Biological Studies

#### 4.2.1. Reagents

Dulbecco’s modified Eagle’s medium (DMEM) was purchased from GE Healthcare Life Sciences, HyClone Laboratories (Logan, UT, USA). The 0.25% trypsin 2.2 mM EDTA lysis buffer was purchased from Corning Life Sciences (VWR International, LLC, Radnor, PA, USA). The 0.25% trypsin and 2.2 mM EDTA lysis buffer were purchased from Corning Life Sciences (VWR International, LLC, Radnor, PA, USA). The phosphate buffer saline (PBS) was purchased from Media Tech, Inc. (Manassas, VA, USA). The Fluoroshield mounting medium, with 6-diamidino-2-phenylindoledihydrochloride (DAPI), was purchased from Abcam (Cambridge, MA, USA). The mitochondrial membrane potential apoptosis kit, with Mitotracker™ Red Annexin V Alexa Fluor^®^ 488, was purchased from Thermo Fisher Scientific (Richard St, Wayne, MI, USA. Dimethylthiazol-2-yl-2, 5-diphenyltetrazolium bromide (MTT) was purchased from Calbiochem EMD Millipore (Billerica, MA, USA). Propidium iodide (PI) was purchased from Life Technologies (Eugene, Oregon, USA). 2′,7′-Dichlorofluorescin diacetate powder was purchased from Sigma Aldrich (St. Louis, MO, USA 63146).

#### 4.2.2. Cell Culture

The human breast cancer cell line, BT-20 and the colorectal cancer cell lines, HCT116, SW620, and HT29, were a kind gift from the late Dr. Gary Kruh (University of Illinois at Chicago, Chicago, IL, USA). The cells were grown in a culture flask as an adherent monolayer in a culture media containing DMEM, supplemented with 4.5 g of glucose, 10% fetal bovine serum (FBS), and 1% penicillin/streptomycin), in an incubator, at 37 °C with 5% CO_2_ and relative humidity of 95%.

#### 4.2.3. MTT Assay

The MTT assay was performed as previously described [38]. Briefly, the cells were seeded in 96-well plates at a density of 3000 cells/well. The cells were incubated for 24 h prior to incubation with the test compounds. The cells were incubated 72 h with either vehicle (DMEM media supplemented with 10% FBS and 1% penicillin and streptomycin; negative control) or the test compound (1–100 μM). Concentrations of 1, 10, or 100 μM were evaluated in the preliminary screening of the compounds in BT-20 and HCT116 cells. The CRC cell lines, HCT116, HT29 and SW620, were incubated with 1, 2, 4, 8, 16, 50, or 100 μM of **DW-8** for 72 h. Following incubation with the test compounds, 20 μL of a 4 mg/mL solution of the MTT reagent was added to each well and the cells were incubated for 3 h at 37 °C. The media was aspirated and the formed formazan crystals were dissolved in 150 μL of DMSO. The wells in the plate were analyzed using a BioTek™ Synergy™ H1Multi-Mode Reader (Winooski, VT, USA) at a wavelength of 570 nm.

#### 4.2.4. Cell Cycle Analysis

The cell cycle analysis was conducted using flow cytometry with propidium iodide (PI) staining to determine the distribution of the cells in the phases of the cell cycle, as previously described [39]. Briefly, SW620 cells were seeded in a 6-well plate at a density of 250,000 cells/well. After incubation overnight, the cells were incubated with vehicle, 1, or 30 μM of **DW-8** for 24 h. Subsequently, the cells were trypsinized with 0.05% trypsin and 2.21 mM EDTA, washed once with phosphate buffer, and re-suspended in 1 mL of ice-cold PBS. The DNA of the cells was stained with 100 µL of the 50 µg/mL PI stock solution and incubated on ice for at least 15 min. The distribution of the cells in the cell cycle phases (SubG1, G1, S, G2) was determined using a BD FACS Canto™ flow cytometer (BD Biosciences, Becton-Dickinson, San Jose, CA, USA). Finally, the data were analyzed using FCS Express 7 cytometry (De Novo software, Pasadena, CA, USA).

#### 4.2.5. Detection of Reactive Oxygen Species (ROS)

SW620 cells were seeded in a 6-well plate at a density of 200,000 cells/well and incubated overnight. The next day, the cells were incubated either in a vehicle or **DW-8** (10 µM) or paclitaxel (0.5 µM; positive control) for 24 h. Subsequently, the cells were incubated with 2′,7′-dichlorodihydrofluorescein diacetate (DCFDA) (3 µM) for 30 min. The cells were washed three times with PBS. Finally, the level of ROS was determined based on the fluorescence level of the oxidized DCFDA dye with a green channel, using BioTek Citation 7™ at 20× magnification.

#### 4.2.6. Nuclear Staining

SW620 cells were seeded at a density of 200,000 cells per well in 6-well plates with microscopic cover slips and incubated overnight. The following day, the cells were incubated with vehicle or 10 and 30 µM of **DW-8**. After 24 h of incubation, the cells were fixed using 4% paraformaldehyde (1 mL/well) for 15 min. The cells were washed gently with PBS. The microscopic cover slips were taken out and mounted in glass slides with the cells facing towards the glass slide and placed in one drop of 4′,6-diamidino-2phenylindol (DAPI) (1 µM). The edges of the cover slip were secured using nail paint. The fluorescence of the stained nuclei was imaged using the blue channel (for DAPI, the absorption maximum was 358 nm, and the emission maximum was 461 nm) in BioTek Citation 7™ at 20× magnification.

#### 4.2.7. Determination of the Mitochondrial Membrane Potential and the Detection of Apoptosis

The effect of **DW-8** on mitochondrial membrane potential and apoptosis was determined using MitoTracker^®^ Red and Alexa Fluor 488 annexin V kits (Molecular Probes Inc., Eugene, OR, USA), respectively, using flow cytometry, as previously described [40]. SW620 cells were seeded in 6-well plates at a density of 300,000 cells/well and incubated with the vehicle, 10 or 30 µM of **DW-8** for 24 h. The cells were detached using the TrypLE reagent (Thermofisher Scientific, Waltham, MA, USA) and collected in 15 mL tubes. Subsequently, 4 µL of the MitoTracker^®^ Red working solution (10 µM) was added to 1 mL of the media with the harvested cells and incubated for 30 min at 37 °C with 5% CO_2_. The cells were washed once with ice-cold phosphate-buffered saline (PBS) to remove the excess MitoTracker^®^ Red. Annexin was added in two steps. First, cells were re-suspended in 100 µL of 1× annexin-binding buffer and 5 µL of Alexa Fluor^®^ 488 Annexin V was added, followed by incubation at room temperature for 15 min. Second, 400 µL of 1× of the Annexin binding buffer was added to the suspended cells, followed by gentle mixing and the cells were placed on ice. The cells were immediately analyzed by flow cytometry to detect the fluorescence excitation/emission maximum (Alexa Fluor^®^ 488 Annexin V: 499/521 nm; MitoTracker^®^ Red: 579/599 nm) using a flow cytometer (BD FACS Canto™ from BD Biosciences, (Becton-Dickinson, San Jose, CA, USA). These data were analyzed using FCS Express 7 Cytometry (De Novo software, Pasadena, CA, USA).

#### 4.2.8. Determination of Cell Viability Using the IncuCyte™ Cytotox Green Assay

The IncuCyte™ Cytotox Green assay was used to detect non-viable cells as previously described [33]. SW620 cells were seeded at a density of 1000 cell/well in 96-well plates. After 24 h of incubation, the cells were incubated with the vehicle or **DW-8** (3, 10, or 30 μM), followed by the addition of 0.25 μM of the Cytotox Green reagent. The cells were incubated in the IncuCyte Zoom live cell imaging apparatus and the green fluorescence was recorded at various time points for up to for 72 h. The IncuCyte^®^ integrated analysis software was used to analyze the results (IncuCyte ZOOM version 2016A, Essen BioScience, Ann Arbor, MI 48108, USA)

#### 4.2.9. Western Blot

Western blot assays were used to identify the important molecular markers of apoptosis as previously described [40]. Briefly, SW620 cells incubated with **DW-8** (10 μM, 30 μM for 24 h), the negative control (0 μM) and PTX (0.5 μM), were lysed using lysis buffer, M-PER™ (ThermoFisher, Rockford, IL, USA), along with a protease inhibitor cocktail containing Aprotinin, Destatin, E-64, Leupeptin, and Pepstatin A (Sigma-Aldrich Life Science, St. Louis, MO, USA). The protein concentration of the cell lysates was quantified using the bicinchoninic acid (BCA) assay (G-Biosciences, St. Louis, MO, USA) [41]. The lysates were loaded onto a polyacrylamide gel for electrophoretic separation. The proteins were transferred from the gel to a polyvinylidene difluoride (PVDF) membrane. The membranes were blocked using 5% milk in Tris-buffer saline Tween 20 (TBST) for 1 h and incubated with primary antibodies (1:1000) at 4 °C with gentle shaking overnight. The membrane was washed with TBST and incubated with horseradish peroxidase labeled (HRP) secondary antibodies (1:4000) for 1 h. Subsequently, the membranes were washed and developed using enhanced chemiluminescent (ECl) substrates, SuperSignal™ Pico/Femto (ThermoFisher, Rockford, IL, USA). ImageJ software was used for the quantification of blots. Protein was normalized with beta-actin.

#### 4.2.10. Statistical Analysis

GraphPad Prism 5 (GraphPad Software, Inc., La Jolla, CA, USA) was used in the statistical analysis of the data. The IC_50_ values obtained from the MTT assay were analyzed using the unpaired *t*-test with Welch’s correction. Data from the MitoTracker^®^ Red and Alexa Fluor 488 Annexin V assay, cell cycle, and western blot quantification were analyzed using two-way ANOVA, followed by Bonferroni’s post-hoc analysis. The green fluorescence of the ROS assay was analyzed using the unpaired *t*-test with Welch’s correction.

## Figures and Tables

**Table 1:** The efficacy of 4-anilinoquinazoline compounds (**DW 1**–**10**) in the colorectal cancer cell line, HCT116 and the breast cancer cell line, BT-20.

DW Compounds	R	IC_50_ (µM)	
		HCT116	BT-20
**1**	H	64.44 ± 0.90	65.94 ± 7.89
**2**	3-CF_3_	40.05 ± 4.65	30.06 ± 7.84
**3**	3,4-dimethyl	75.77 ± 10.98	88.50 ± 30.65
**4**	4-COCH_3_	>100	>100
**5**	3,4-dimethoxy	>100	>100
**6**	4-OCH_3_	>100	>100
**7**	4-NHCOCH_3_	>100	>100
**8**	3-Cl, 4-F	8.50 ± 2.53	11.69 ± 3.73
**9**	4-CF_3_	44.70 ± 5.63	39.46 ± 3.74
**10**	4-OCF_3_	49.20 ± 7.27	45.61 ± 3.31

The MTT assay was performed as described in the material and methods section. The IC_50_ values are reported as the mean ± SD of three independent experiments performed in triplicate.

## Data Availability

Not applicable.

## Sample Availability

Samples of the compounds are available from the authors upon request.
